# When should the nephrologist think about genetics in patients with glomerular diseases?

**DOI:** 10.1093/ckj/sfaf044

**Published:** 2025-02-13

**Authors:** Roser Torra, Xoana Barros, Montserrat Díaz-Encarnación, Leonor Fayos, Mónica Furlano, Melissa Pilco, Marc Pybus, Amir Shabaka, Elizabeth Viera, Elisabet Ars

**Affiliations:** Nephrology Department, Fundació Puigvert, Institut de Recerca Sant Pau (IR-Sant Pau), Departament de Medicina, Universitat Autònoma de Barcelona, Barcelona, Spain. RICORS2040; Nephrology Department, Fundació Puigvert, Institut de Recerca Sant Pau (IR-Sant Pau), Departament de Medicina, Universitat Autònoma de Barcelona, Barcelona, Spain. RICORS2040; Nephrology Department, Fundació Puigvert, Institut de Recerca Sant Pau (IR-Sant Pau), Departament de Medicina, Universitat Autònoma de Barcelona, Barcelona, Spain. RICORS2040; Nephrology Department, Fundació Puigvert, Institut de Recerca Sant Pau (IR-Sant Pau), Departament de Medicina, Universitat Autònoma de Barcelona, Barcelona, Spain. RICORS2040; Nephrology Department, Fundació Puigvert, Institut de Recerca Sant Pau (IR-Sant Pau), Departament de Medicina, Universitat Autònoma de Barcelona, Barcelona, Spain. RICORS2040; Nephrology Department, Fundació Puigvert, Institut de Recerca Sant Pau (IR-Sant Pau), Departament de Medicina, Universitat Autònoma de Barcelona, Barcelona, Spain. RICORS2040; Molecular Biology Laboratory, Fundació Puigvert, Institut de Recerca Sant Pau (IR-Sant Pau), Universitat Autònoma de Barcelona, Barcelona, Spain. RICORS2040; Nephrology Department, Hospital Universitario la Paz, Madrid, Spain. RICORS2040; Nephrology Department, Fundació Puigvert, Institut de Recerca Sant Pau (IR-Sant Pau), Departament de Medicina, Universitat Autònoma de Barcelona, Barcelona, Spain. RICORS2040; Molecular Biology Laboratory, Fundació Puigvert, Institut de Recerca Sant Pau (IR-Sant Pau), Universitat Autònoma de Barcelona, Barcelona, Spain. RICORS2040

**Keywords:** FSGS, genetic, genetic testing, glomerular, proteinuric

## Abstract

This review discusses the significance of genetics in diagnosing glomerular diseases. Advances in genetic testing, particularly next-generation sequencing, have improved the accessibility and accuracy of diagnosing monogenic diseases, allowing for targeted gene panels and whole-exome/genome sequencing to identify genetic variants associated with glomerular diseases. Key indicators for considering a genetic cause include the age of onset, extrarenal features, family history, and inconclusive kidney biopsy results. Early-onset diseases, for instance, have a higher likelihood of being genetically caused, while extrarenal manifestations can also suggest an underlying genetic condition. A thorough family history can reveal patterns of inheritance that point to monogenic causes, although complexities like incomplete penetrance, skewed X inactivation and mosaicism can complicate the assessment. Also, autosomal recessive conditions imply asymptomatic parents, making genetic suspicion less likely, while *de novo* mutations can occur without any family history, further obscuring genetic assessment. Focal segmental glomerulosclerosis (FSGS) is characterized by podocyte injury and depletion, presenting in various forms, including primary, genetic, and secondary FSGS. Accurate classification of FSGS patients based on clinical and histological features is essential for guiding treatment decisions, optimizing therapeutic plans, avoiding unnecessary immunosuppression, and predicting relapse risk after kidney transplantation. Overall, a clinicopathological approach, enriched by genetic testing, offers a precise framework for diagnosis and management in glomerular diseases. Future directions for research and clinical practice include potential advancements in genetic testing and personalized medicine, which could further improve diagnostic precision and individualized treatment strategies.

## INTRODUCTION

Kidney disease represents a major global health challenge, with its prevalence on a steady rise, now ranking as the seventh leading cause of mortality worldwide. Approximately 700 million individuals globally are affected by chronic kidney disease (CKD). A recent analysis from the European Renal Association registry revealed that in 2019, among prevalent patients receiving kidney replacement therapy (KRT), inherited kidney diseases (IKDs) and congenital anomalies of the kidney and urinary tract (CAKUTs) accounted for 18.5% of cases, while glomerulonephritis was responsible for 18.7%. Notably, IKD-CAKUT emerged as the leading cause of kidney failure in women (21.6%) [1]. However, these figures are likely underestimated, as they reflect 2019 data and a pan-European perspective. Genetic testing availability varied across Europe at that time (and still does) and has since improved, suggesting that a portion of patients diagnosed with ‘glomerulonephritis’ or ‘other diseases’ may, in fact, have underlying genetic conditions. This would position IKD-CAKUT as the most prevalent cause of KRT.

A key reason for the higher prevalence of IKD-CAKUT is that these patients often present with fewer severe comorbidities, tend to be younger, and have longer survival rates compared with others on KRT. Beyond demographic considerations, the advent of next-generation sequencing (NGS) technologies has made genetic testing more accessible and affordable, providing an invaluable tool for diagnosing glomerular diseases.

Today, genetic testing options range from targeted gene panels, which focus on specific exonic regions linked to glomerular diseases, to more comprehensive approaches like exome sequencing, which examines all protein-coding regions, and whole-genome sequencing (WGS), which covers the entire genome. Recent technological advancements, particularly the development of long-read sequencing, have enhanced diagnostic precision. Short-read sequencing, while highly accurate and cost-effective for identifying small variants such as single-nucleotide variants and small indels, struggles with detecting large structural variants or long repetitive sequences. However, it is worth noting that short-read WGS shows a higher sensitivity for detecting structural variants than whole-exome sequencing (WES) or other targeted approaches, as it can detect the intergenic breakpoints of balanced structural variants, such as inversions. In contrast, long-read sequencing overcomes these limitations by reading longer DNA fragments, which also enables the detection of complex genomic rearrangements, although it remains costlier at present [2]. These innovations are pushing genetic testing toward more comprehensive, precise, and personalized diagnostics.

**Figure 1:**
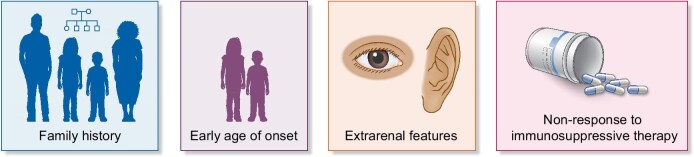
Key features to elicit a request for a genetic test for glomerular disease.

In this review we will explore the key indicators (Fig. 1) that suggest an inherited glomerulopathy, address the challenges associated with genetic testing, and highlight the benefits of achieving a precise diagnosis.

## FEATURES SUGGESTING A GLOMERULAR DISEASE OF GENETIC ORIGIN

### Age of onset

Early onset of glomerular disease should prompt suspicion of a genetic origin, although considerable variability exists, reflecting the broad phenotypic spectrum observed across different conditions. Recognizing this variability is essential for guiding clinical decisions regarding the need and timing of genetic testing.

In recent years, the number of genes implicated in genetic steroid-resistant nephrotic syndrome (SRNS) and focal segmental glomerulosclerosis (FSGS) has expanded significantly. Multiple guidelines for genetic screening have been proposed, particularly for congenital and infantile cases, where the likelihood of identifying a monogenic cause is higher [3]. The prevalence of pathogenic variants has been shown to inversely correlate with the age of onset. For example, a 100% detection rate was reported for congenital nephrotic syndrome (with most pathogenic variants found in the *NPHS1* gene), 57% in infantile onset, and 24%–36% in childhood and adolescent onset (mainly *NPHS2*) in a cohort of 125 patients [4, 5]. Although the diagnostic yield has traditionally been lower for adult-onset cases, disease-causing pathogenic variants have also been detected, with an estimated 8%–14% of adult-onset SRNS/FSGS, including both familial and sporadic cases [6–8].

Pathogenic variants in non-collagen genes tend to be found in patients with proteinuria onset before the age of 30, while heterozygous pathogenic variants in collagen genes may manifest even beyond the fifth decade of life. FSGS is a well-known pathological finding in Alport syndrome, which is marked by a wide phenotypic spectrum and variable age of onset depending on the mode of inheritance [9, 10]. Variants in collagen genes (*COL4A3, COL4A4, COL4A5*) are among the most common disease-causing variants identified in adult patients with sporadic SRNS/FSGS, accounting for 44%–56% of cases [9, 10].

The advent of high-throughput sequencing technologies in adult CKD cohorts has reinforced the importance of achieving an accurate diagnosis, even in adulthood. A study of 1623 CKD patients, categorized into specific clinical disease groups with a median age of 55 years, found a diagnostic yield of 16.9% among those with proteinuric diseases suggestive of primary glomerulopathy. Variants in *COL4A3*/*COL4A4*/*COL4A5* were responsible for 31.8% of positive cases, while high-risk *APOL1* genotypes were identified in 45.5% of cases [11]. In a recent Spanish cohort of 818 adult patients under 45 years with CKD stage 4–5 of unknown origin, the two leading diagnosed conditions were Alport syndrome spectrum (35%) and genetic podocytopathies (19%) [12].

Previous research from our group demonstrated that seven genes accounted for 66% of positive genetic tests among individuals with CKD onset before 30 years of age. Interestingly, five of these genes (*COL4A3*/*COL4A4*/*COL4A5, PKD1*, and *PKD2*) were also among the six most frequently detected in an adult cohort studied by Groopman *et al*., with *PKHD1* and *HNF1B* more common in the younger cohort, while *UMOD* was more prevalent in older individuals [5, 9].

IKD and CAKUT are the leading causes of kidney failure in patients younger than 20 years (41% of cases), but their incidence peaks in patients over 45, mainly due to autosomal dominant polycystic kidney disease (ADPKD) [1].

Understanding that genetic kidney diseases can present at any age emphasizes the need for greater awareness and the importance of genetic testing across all age groups. However, given that the prevalence of genetic kidney diseases is higher among younger patients, it is particularly important to consider genetic causes in children and young adults with CKD as well as in patients with CKD of unknown etiology, especially those wait-listed for kidney transplantation for whom a living-related donor is being considered.

### Extrarenal features

Glomerular diseases of genetic origin frequently involve multiple systems and are not confined to the kidneys. Notably, monogenic disorders with recessive inheritance patterns often result in rare syndromes that strongly suggest an underlying genetic condition. Table 1 offers a detailed overview of the extrarenal manifestations commonly associated with genetically driven glomerular diseases (complementopathies beyond atypical hemolytic uremic syndrome (aHUS) are not included due to its complex inheritance). Some key examples are discussed below.

**Table 1:** Genes associated with glomerular disorders classified according to the part of the of the glomerulus/nephron involved.

Location of anomaly	Gene	Age of onset	Inheritance pattern	Extrarenal features	Suggestive biopsy features
**Podocyte/slit diaphragm** [46]	*NPHS1* (nephrin) [47, 48]	Congenital, neonatal, infantile.	AR	None.	LM: FSGS Mesangial hypercellularity. Hyperlobulated capillary tufts. EM: podocyte fusion. Irregular podocyte foot processes [49, 50].
	*NPHS2* (podocin) [51–53]	Varies from congenital forms to young adult.	AR	None.	LM: FSGS, DMS EM: podocyte fusion. Irregular podocyte foot processes [28].
	*CRB2* (crumbs family member 2) [54, 55]	Congenital	AR	May present with elevated maternal alpha-fetoprotein levels, ventriculomegaly, hydrocephaly, cardiac and ocular defects. Also, only a renal form with nephrotic proteinuria has been described until adolescence.	LM: FSGS
	*CD2AP* (CD2 associated protein) [56, 57]	Childhood, young adulthood	AD/AR	None.	LM: FSGS, EM: effacement of the podocyte processes. (PMID:
	*FAT1* [58]	Childhood to young adulthood	AR	Neurologic involvement; dysmorphic features, colobomatous microphthalmia, hematuria.	LM: FSGS, tubular ectasia. EM: effacement of the podocyte processes
	*TRPC6* (transient receptor potential cation channel, subfamily C, member 6) [59, 60]	Young adulthood	AD	None.	LM: FSGS
**Podocyte cytoskeleton:** **actin regulation** [46]	*ARHGAP24* (Rho GTPase-activating protein 24) [61]	Adolescence	AD	None.	LM: FSGS
	*ARHGDIA* (Arhgdia) [62, 63]	Congenital, early childhood.	AR	Rare: intellectual disability, neurosensorial deafness, seizures and cortical blindness.	LM: FSGS, DMS [64]
	*KANK1, KANK2, KANK4* (kidney ankyrin repeat containing protein) [65]	Infantile	AR	N/A	LM: FSGS
	*DLC1, CDK20, TNS2, ITSN1, ITSN2* [66]	Early onset to childhood	AR	None. Some of these patients were partially sensitive to steroid or cyclosporine A treatment.	LM: FSGS EM: effacement of the podocyte processes.
	*RHOA, RAC1, CDC42* [67]	Early onset	AR	None.	LM: FSGS
	*MAGI2* (membrane-associated guanylate kinase, WW, PDZ domain containing 2) *TNS2* (tensin-2) [68, 69]	Congenital, infantile	AR	None.	LM: FSGS
**Podocyte cytoskeleton:** **actin binding** [46]	*ACTN4* (α-actinin-4) [70, 71]	Young adult	AD	None.	LM: FSGS
	*ANLN* (anillin) [72]	Young adult, childhood.	AD	None.	LM: FSGS
	*AVIL* (advillin) [73, 74]	Infantile	AR	Neurological symptoms (microcephaly, deafness, development delay, retinal dystrophy).	LM: FSGS
	*MYH9* (non-muscle myosin heavy chain IIA) [75, 76]	Young adulthood	AD	Epstein–Fechtner syndrome: Macrothrombocytopenia, leukocytes with cytoplasmic inclusion bodies (Döhle-like bodies), sensorineural deafness, cataracts at an early age.	LM: FSGS EM: focal and segmental effacement of podocytes and loss of the interpodocyte slit diaphragm. Also, reported irregular thickening and splitting, focal attenuation, and basket weave appearance of the GBM.
	*INF2* (inverted formin-2) [77, 78]	Adolescence, young adulthood	AD	Charcot–Marie–Tooth syndrome.	LM: FSGS
	*MYO1E* (myosin-1E) [79, 80]	Childhood	AR	None.	LM: FSGS
	*PLCE1* (phospholipase C epsilon-1) [81, 82]	Childhood	AR	None.	LM: FSGS, DMS
**Podocyte** **cytoskeleton: microtubule regulation** [46]	*WDR73* (WD repeat domain-73), *WDR4* (WD repeat domain-4) [83, 84]	Congenital to childhood onset	AR	Galloway–Mowat syndrome (GAMOS).	LM: FSGS, DMS
**Podocyte:** **tRNA regulation of protein synthesis** **KEOPS complex** [85]	*TP53RK* (TP53 regulating kinase), *TPRKB* (TP53RK binding protein), *GON7, YRDC, OSGEP* (O-sialoglycoprotein endopeptidase) [85, 86]	Congenital to childhood onset	AR		
	*LAGE3* (L-antigen family member 3) [86]	Congenital to childhood onset	XL		
**Podocyte foot process:** **CoQ10 biosynthesis (foot process)** [46]	*COQ8B* (previously named *ADCK4*), *COQ2, COQ6* [87, 88]	Childhood, adolescence to young adult.	AR	*COQ8B*: non-extrarenal symptoms. Is the less severe. *COQ2*: retinopathy, mypathy, neurological and development issues. *COQ6*: neurosensorial deafness, neurological symptoms (seizures, encephalopathy, development delay, sometimes retinopathy).	LM: FSGS
	*MTTL1* (mitochondrially encoded tRNA leucine 1) [89]	Childhood Some cases in young adulthood when presents as isolated FSGS	Mitochondrial	MELAS syndrome.	LM: FSGS
	*PDSS2* (decaprenyl diphosphate synthase subunit 2) [90]	Congenital, neonatal	AR	Leigh syndrome deficit CoQ10 with neurological symptoms.	LM: FSGS
**Podocyte foot process:** **nucleus** [46]	Nucleoporins: *NUP93, NUP205, NUP85, NUP107, NUP133, NUP60* *XPO5* [91, 92]	Childhood to adolescence	AR	None. *NUP107* and *NUP 133* have been identified in patients with neurological symptoms as part of Galloway–Mowat syndrome.	LM FSGS, DMS *NUP93* reported in collapsing FSGS.
	*LMX1B* (LIM homeobox nuclear transcription factor 1B) [93, 94]	Childhood to young adulthood	AD	Nail–patella syndrome Some missense variants cause isolated FSGS.	LM FSGS EM: podocyte foot processes effacement.
	*SMARCAL* (SWI/SNF2-related, matrix-associated, actin-dependent regulator of chromatin, subfamily a-like 1) [95, 96]	Infantile	AR	Spondyloepiphyseal dysplasia, T-cell immunodeficiency. 50% of patients may also present hypothyroidism, episodic cerebral ischemia; a few patients may have bone marrow failure.	LM: FSGS
	*WT1* (Wilms tumor 1) [97, 98]	Childhood to adolescence onset	AD	Frasier syndrome, Denys–Drash syndrome.	LM: FSGS
**Podocyte foot process:** **lysosome** [46]	*SCARB2* (scavenger receptor class B member 2) [99, 100]	Late childhood or adolescence	AR	*SCARB2*-related action myoclonus–renal failure syndrome: causes progressive myoclonic epilepsy (PME). Renal manifestations (SRNS) often precede neurological symptoms, and sometimes only neurological presentation exists without renal involvement.	LM: FSGS
**Podocyte foot process:** **S1P metabolism** [46]	*SGPL 1* (sphingosine-1-phosphate lyase) [101, 102]	Congenital	AR	Few cases described, some findings were adrenal insufficiency, hypothyroidism, immunodeficiency, neurological symptoms, ichthyosis.	LM: FSGS, DMS
**Podocyte structure preservation** **(other genes)**	*PTPRO* (receptor-type tyrosine protein phosphatase-O), also known as *GLEPP1* [103].	Childhood to adolescence	AR	None.	LM: FSGS
	*EMP2* (epithelial membrane protein 2) [104, 105]	Childhood	AD/AR	None.	LM: FSGS
	*CUBN* (cubilin) [106–108]	Childhood	AR	Imerslund–Gräsbeck syndrome; some cases reported in patients with isolated FSGS (N-terminal variants) Cubilin also causes isolated proteinuria with normal renal function (C-terminal variants).	LM: FSGS
	*KIRREL1* [109]	Childhood to adolescence	AR	None.	LM: FSGS
**Glomerular basement membrane (GBM)**	*COL4A5* [16, 110]	Childhood to adulthood	XL	Alport syndrome Ocular: anterior lenticonus, retinopathy (retinal staining), maculopathy. Auditive: high tone sensorineural hypoacusia.	LM: non-specific, FSGS, interstitial foam cells. Immunostaining for type IV collagen alpha chains can confirm absence of alpha 5 type IV collagen in males. EM: irregular thickening of GBM, GBM split or lamellated. Extensive podocyte foot process effacement is observed, podocyte protrusions invading the GBM [111].
	*COL4A3, COL4A4* [4, 112]	Childhood to adulthood	AR/AD	AR: same as *COL4A5* AD: rarely extrarrenal manifestation reported (PMID: 33838161).	AR: same as *COL4A5* AD: LM: unspecific, GEFS and sometimes mesangial expansion with no staining or unspecific staining. EM: irregular thickening of GBM, sometimes podocyte foot process effacement [111, 113].
	*ITGA3* (alpha-3 integrin) [114]	Congenital	AR	Congenital nephrotic syndrome, interstitial lung disease, and epidermolysis bullosa.	LM: FSGS
	*ITGB4* (beta-4 integrin)	Congenital	AR	Epidermolysis bullosa, pyloric atresia, occasionally aplasia cutis.	LM: FSGS
	*LAMB2* (laminin subunit beta 2) [115, 116]	Congenital to childhood	AR	Pierson syndrome.	LM: FSGS, DMS EM: severe podocyte foot process effacement and irregular thickening of the GBM. IF: absent beta2 laminin staining.
	*LAMA5* [117, 118]	Childhood to young adults	AD	Some pulmonary defects described.	LM: FSGS ME: Effacement of podocyte foot processes.
**Endothelium** ^a^	*CFH, C3, CFI, CFB* (cell surface and fluid phase regulation of CAP) and *MCP* (cell surface regulation of CAP). ***Search for variants and hybrid genes***^b^ [119–121]	High variability in the age of onset, mostly childhood	Heterozygous/homozygous/compound heterozygous PMID: 23307876	TMA: microangiopathic hemolytic anemia (high LDH serum levels, low haptoglobin, presence of schistocytes in peripheral blood smear, negative Coombs test). Thrombocytopenia. Other organs could be affected: central nervous system.	**Acute lesions:** LM: presence of fibrin thrombi in glomerular capillaries, arterioles, subendothelial areas, and in the mesangium. mesangiolysis, endothelial swelling, corrugation of the glomerular basement membrane (GBM) IF: thrombi stain positive for fibrinogen. Non-specific staining for IgM in glomeruli, and less frequently C3 and IgG. **Chronic lesions:** LM: double contour of glomerular capillary walls (membranoproliferative-like pattern), intimal thickening and concentric lamination (onion skin lesions) of the arterioles. FSGS [122].
	*DGKE* (diacylglycerol kinase ε) is expressed in endothelium, platelets and podocytes [37]	Early onset (<1 year old)	Homozygous/compound heterozygous		
	*THMD* (thrombomodulin) [123, 124]	Mostly childhood	Heterozygous		
**Other genetic diseases presented as glomerulopathy**	*GLA* (α-galactosidase A) [125, 14]	Childhood (males) to adulthood (mostly females)	XL	Classic Fabry disease: neurological (distal pain, acroparesthesias, heat intolerance, hearing loss, tinnitus), ischemic brain disease. Gastrointestinal: nausea, vomiting, diarrhea, postprandial bloating and early satiety, weight loss. Skin: angiokeratomas, hypohidrosis. Eyes: corneal opacities, cornea verticillate, vascular retinal tortuosity. Heart: arrythmias, valvular insufficiency, hypertrophic myocardiopathy. Non-classic Fabry disease: delayed onset, sometimes only one organ affected. Cardiac involvement.	LM: FSGS, vacuolated appearance of podocytes, parietal epithelial cells, and distal tubular epithelial cells. Hyaline-like material accumulates in media of arteries and arterioles (Fabry arteriopathy) and sometimes in mesangial regions. EM: presence of zebra bodies. Podocyte foot process effacement [126].
	*TTC21B* (tetratricopeptide repeat domain-21) [127, 128]	Childhood to adolescence	AR	None.	LM: FSGS with tubulointerstitial lesion associated.
	*PAX2* (paired box 2) [129–131]	Adolescence to young adulthood	AD	Oculopapilar syndrome CAKUT associated with extrarenal manifestations in central nervous system, ocular, and sensorineural hearing loss.	LM: FSGS
	*EYA1* [4]	Childhood. Adulthood when FSGS isolated	AD	Branchio-oto-renal syndrome.	LM: FSGS

CAP, complement alternative pathway; DMS, diffuse mesangial sclerosis; EM, electron microscopy; FSGS, focal segmentary glomerulosclerosis; GBM, glomerular basement membrane; LM, light microscopy; SRNS, steroid-resistant nephrotic syndrome; TMA, thrombotic microangiopathy.

^a^Genes of the complement cascade that cause TMA are not always expressed at the endothelium but the mechanism of the lesion in the glomeruli lies in the endothelium.

^b^Rare variants in these genes are present in some healthy individuals (∼10%); the enrichment of rare variants in one or several complement genes and the presence of risk polymorphisms suggest the role of CAP in the pathogenesis of the disease and explains the variability in the penetrance of the disease [120].

**Fabry disease** is an X-linked lysosomal storage disorder caused by pathogenic variants in the *GLA* gene, leading to the accumulation of globotriaosylceramide (Gb-3) throughout the body. Ophthalmologically, this results in cornea verticillata (subepithelial spiral opacities that do not impair vision), posterior cataracts, and tortuosity of the retinal vessels. In the integumentary system, Gb-3 deposits lead to angiokeratomas and hypohidrosis or anhidrosis, contributing to heat intolerance. The gastrointestinal system may be affected by recurrent abdominal pain, nausea, and diarrhea due to vascular and autonomic dysfunction. Additionally, patients often experience progressive sensorineural hearing loss, which is sometimes accompanied by tinnitus and vertigo. These features compound the primary organ involvement affecting the kidneys, heart, and brain [13, 14].

**Alport syndrome** is often associated with high-frequency sensorineural hearing loss, anterior lenticonus, anterior subcapsular cataracts, and punctate keratopathy [15, 16].

**Frasier syndrome** results from pathogenic variants in the *WT1* gene, which encodes a transcription factor involved in renal and gonadal development. Systemic manifestations include male pseudohermaphroditism, ambiguous genitalia, hypospadias, cryptorchidism, gonadoblastoma, and, in rare cases, nephroblastoma (Wilms’ tumor) [17, 18].

**Nail–patella syndrome**, caused by pathogenic variants in the *LMX1B* gene and inherited in an autosomal dominant manner, presents with a range of extrarenal features such as hypoplastic or absent patellae, dystrophic nails, elbow dysplasia, iliac horn dysplasia, and open-angle glaucoma. Interestingly, some individuals with this condition may exhibit no renal symptoms at all [19, 20].

The presence of such extrarenal manifestations should raise the suspicion of an underlying genetic condition and warrant the consideration of genetic testing.

### Family history

When evaluating the potential for a monogenic cause of glomerular disease, a detailed family history is a critical tool. However, several genetic factors can complicate the family history, making the monogenic suspicion less apparent. Complex inheritance patterns, incomplete penetrance, and mechanisms such as X-inactivation can obscure how glomerular diseases manifest across different family members [21].

Monogenic glomerular diseases can follow various inheritance patterns. For instance, **autosomal Alport syndrome** is inherited in a semi-dominant manner, where individuals with biallelic pathogenic variants exhibit more severe disease than those with a single, monoallelic variant, who tend to experience milder symptoms. In the autosomal dominant form of the disease, where only one mutated allele is needed to cause illness, the condition can appear across multiple generations, with very variable severity. Additionally, incomplete penetrance can result in the presence of the mutated allele in asymptomatic family members, giving the impression that the disease skips a generation.

A more severe form of **Alport syndrome** is X-linked, where males typically present more severe symptoms, and females may display milder disease due to X-inactivation. X-inactivation occurs when one copy of the X chromosome is randomly inactivated in early female embryonic cells. However, skewed inactivation may favor either the wild-type or mutated allele, influencing disease expression. In such cases, females can exhibit varying severity depending on which X chromosome is inactivated in the majority of cells.

Alport syndrome presents yet another pattern of inheritance, as exemplified by **autosomal recessive Alport syndrome**. In autosomal recessive conditions, affected family members are typically siblings, while parents are asymptomatic carriers. As a result, a family history of kidney disease may be absent, making the suspicion of a genetic condition less likely. However, because autosomal recessive diseases are often severe, early presentation of the disease can raise suspicion of a genetic cause. In this case parents may not be asymptomatic and can be considered to have **autosomal dominant Alport syndrome.**

Another complicating factor are ***de novo* pathogenic variants**, which arise spontaneously either in a parental germ cell or during embryonic development. *De novo* variants can obscure genetic suspicion, as they occur without any prior family history, making it challenging to identify a genetic basis for the disease. Additionally, *de novo* cases may exhibit mild clinical features due to **mosaicism**, where the disease-causing variant is present in only a proportion of cells. Mosaicism must be considered during reproductive genetic counseling for the healthy parents of a child with a *de novo* variant in a gene associated with an autosomal dominant glomerular disease. While parents may be reassured that the *de novo* variant poses no risk of recurrence in future children, there is still a possibility of having another affected child due to germinal restricted mosaicism, which may not be detectable by conventional blood-targeted genetic testing.

Given these complexities, nephrologists must take a thorough, multi-generational family history—spanning at least three generations—inquiring not only about known kidney diseases but also potential extrarenal features.

### Kidney biopsy

Kidney biopsy (KB) is a cornerstone diagnostic tool in nephrology, with an estimated diagnostic accuracy of 80%. Recent studies indicate that incorporating genetic testing for patients with inconclusive KB results can significantly enhance etiological diagnosis [22–24]. A single-center study examining the use of exome sequencing in routine clinical practice found that it enabled diagnosis in 40% of patients with previously inconclusive KB, with Alport spectrum-related nephropathy being the most frequently identified genetic disorder. Importantly, the diagnostic yield increased to 70% among patients with a family history of kidney disease [22, 23]. The study cohort, composed predominantly of younger patients, limits the generalizability of these findings to older populations. Nonetheless, the higher prevalence of familial kidney disease observed in the genetically solved group compared with the genetically unresolved group reinforces that a positive family history remains a critical factor in diagnosing genetic kidney disease [22–24]. Moreover, the predominant lesions observed in KB aligned with the genetic diagnoses of glomerular and tubulointerstitial diseases. Typical histological findings for glomerular genetic conditions are presented in Table 1; however, for several conditions the histological findings are non-specific.

It is important to note that a recent report estimated that up to 1 in 106 individuals in the general population carry heterozygous pathogenic variants in the *COL4A3* or *COL4A4* gene [25]. However, the pathogenicity assessment of these variants was conducted using Varsome, a semi-automated online prediction tool, which at that time overestimated pathogenicity. This overestimation arose from the inclusion of criteria such as PP2 and PP5, which ClinGen recommended removing from the original ACMG/AMP guidelines due to concerns about double counting and the potential for errors in variant classification [26]. Although a more precise assessment of the frequency of individuals with a pathogenic variant in *COL4A3*/*COL4A4* is needed, these remain the most common pathogenic variants in genes associated with monogenic glomerular diseases. Furthermore, these genes exhibit incomplete penetrance. Taken together, these factors have implications for the rare cases in which genetic testing is performed prior to KB; finding a heterozygous pathogenic variant in *COL4A3* or *COL4A4* alone may not be diagnostic, as the patient might harbor a variant in these genes alongside an undiagnosed glomerulopathy [27].

### Focal segmental glomerulosclerosis

FSGS is a histological pattern of kidney injury that can arise from a variety of underlying causes and mechanisms, all of which share a common event: podocyte injury and depletion. Accurate identification of the etiological process leading to FSGS is crucial for effective treatment [28]. Consequently, a clinicopathological approach has been recommended, classifying FSGS into primary, genetic, secondary, and FSGS of undetermined cause.

Primary FSGS is an immunologically driven disease that typically presents with full-blown nephrotic syndrome of sudden onset. Under electron microscopy, diffuse podocyte foot process effacement affecting >80% of the glomerular capillary surface is observed [29]. These cases exhibit a high recurrence rate after kidney transplantation, which differentiates them from other FSGS types. Recently, anti-nephrin antibodies were identified in a subset of patients with minimal change disease [30] and recurrent FSGS post-transplant [31]. Furthermore, anti-nephrin and anti-slit diaphragm antibodies were observed in kidney biopsies from adult patients with steroid-resistant nephrotic syndrome and FSGS lesions under high-resolution microscopy [32]. These findings support an autoimmune etiology, suggesting susceptibility to immunosuppressive therapy and opening avenues for precision therapies in the future.

Secondary FSGS arises from a variety of etiologies, including conditions that impose excessive stress on the glomerular filtration barrier (maladaptive forms) or directly injure podocytes (due to drugs, infections, etc.). Patients with secondary FSGS may exhibit varying levels of proteinuria but, unlike primary FSGS, typically maintain normal serum albumin levels and do not develop full nephrotic syndrome. Electron microscopy in secondary FSGS shows segmental foot process effacement, usually affecting less than 40% of the glomerular capillary surface. Maladaptive forms often occur in conditions with reduced nephron mass (e.g. low birth weight, renal dysplasia, reflux nephropathy) or increased glomerular filtration rate exceeding glomerular capacity (e.g. obesity, uncontrolled hypertension, sleep apnea, high protein intake). Other causes of secondary FSGS include virus-associated FSGS (e.g. due to HIV, CMV, or parvovirus B19) and drug-induced FSGS (e.g. caused by TOR inhibitors, lithium, pamidronate, anti-VEGF agents), which generally improve with infection resolution or cessation of the causative drug [33].

Genetic FSGS results from pathogenic variants in genes that encode proteins crucial to podocyte structure or function, or the glomerular basement membrane (GBM). Clinical presentation varies, with some cases resembling primary FSGS with full nephrotic syndrome, while others present with proteinuria and normal serum albumin, similar to secondary FSGS. Although genetic forms of FSGS often have childhood onset, the wide phenotypic spectrum means that adult-onset genetic FSGS cannot be ruled out solely based on age.

FSGS of undetermined cause refers to cases without a clear genetic or secondary origin, in the absence of nephrotic syndrome or diffuse foot process effacement on electron microscopy. These cases are thought to result from genetic or secondary causes yet to be identified.

Accurate classification of FSGS patients based on clinical and histological features is essential for guiding treatment decisions, optimizing therapeutic plans, avoiding unnecessary immunosuppression, and predicting relapse risk after kidney transplantation.

The role of genetic testing in adult FSGS cases remains uncertain. The 2021 KDIGO guidelines recommend case-by-case consideration for genetic testing (Table 2) [34]. A positive family history or syndromic features also increase the likelihood of a genetic diagnosis. Genetic testing is also advised for patients with steroid-resistant nephrotic syndrome, as nearly 42% of these cases have an underlying genetic cause, and a prompt diagnosis could enable the discontinuation of immunosuppressive therapy [9]. In patients of African ancestry, *APOL1* genetic risk variants are associated with a significantly increased risk of developing FSGS, and kidneys from *APOL1* high-risk donors have reduced graft survival compared with non-risk donors. Thus, genetic testing in donors of African ancestry is essential to inform both the donor's risk of chronic kidney disease and the recipient's allograft survival [35].

**Table 2:** Criteria of genetic testing for focal segmental glomerulosclerosis (FSGS) from KDIGO 2021 Clinical Practice Guideline for the Management of Glomerular Diseases.

• Cases of strong family history and/or clinical picture suggestive of syndromic disease.
• Aiding in the diagnosis of FSGS of undetermined cause when clinical features are not representative of a particular disease phenotype.
• In steroid and/or immunosuppressant-resistant FSGS, to limit exposure to unnecessary immunosuppression.
• Before kidney transplantation, to determine risk of recurrent disease.
• Risk assessment in living-related kidney donor candidates and/or high suspicion of APOL1 risk variants.
• Prenatal diagnosis.

### Recurrence in kidney transplantation

Unlike glomerular diseases of non-genetic origin, genetic glomerular diseases typically do not recur in the transplant graft, as the new organ does not carry the mutations present in the original damaged kidney. However, there are some exceptions.

Atypical hemolytic uremic syndrome is a condition marked by thrombotic microangiopathy, which leads to hemolytic anemia, thrombocytopenia, and acute kidney injury [36]. The recurrence rate in kidney transplants is notably high, ranging from 50% to 80%, due to genetic defects in the complement system, involving genes like *CFH, CFI, CD46, CFB*, and *C3* [37, 38]. If a family member is considering becoming a kidney donor, it is crucial to exclude the presence of the pathogenic variant in the donor, given the significant risk of recurrence. Prophylactic treatment with eculizumab (a complement inhibitor) may reduce the risk of recurrence [39–41].

In some genetic diseases, an ‘immune’ recurrence may occur after transplantation. Rare examples include X-linked Alport syndrome in males, recessive Alport syndrome, and the nephrotic syndrome of Finnish type. In these rare cases, the recipient's immune system fails to recognize antigens in the graft and produces antibodies against key parts of the nephron, leading to severe kidney damage.

## POSSIBLE RESULTS OF GENETIC TESTING

Genetic testing in glomerular diseases can yield a range of results, each with specific implications for diagnosis and management. A positive result indicates the presence of a pathogenic or likely pathogenic variant in a gene associated with either monoallelic or biallelic disease. This finding suggests a clear monogenic cause for the glomerular disease, especially if supported by strong evidence of gene–disease association, such as that provided by ClinGen, a National Institutes of Health (NIH)-funded resource that defines the clinical validity of gene–disease relationships for precision medicine [42].

Establishing the disease mechanism is also crucial, since some diseases are caused by loss-of-function variants whereas others are caused by gain-of-function variants. Moreover, variants in some genes may be only pathogenic if they impact specific protein domains. For example, the *INF2* gene encodes a formin family protein involved in actin cytoskeleton remodeling, mitochondrial dynamics, and microtubule stabilization. Monoallelic gain-of-function variants in *INF2* affecting the diaphanous inhibitory domain (DID) encoded by exons 2–4 are associated with autosomal dominant FSGS. However, loss-of-function variants or missense variants outside this domain are not disease-causing.

When a genetic test identifies a variant of uncertain significance (VUS), it should be regarded as a non-diagnostic result, since the clinical significance of the variant remains unclear. This uncertainty arises from several factors, including the high prevalence of rare variants across the genome and the incomplete understanding of the functional impact of many variants. Importantly, acting on a VUS can lead to significant hazards, such as misdiagnosis or inappropriate treatment decisions. For instance, a misdiagnosis based on a VUS could lead to unwarranted interventions, such as immunosuppressive therapy or predictive testing in asymptomatic family members, potentially causing harm. These considerations emphasize the importance of treating a VUS as a hypothesis rather than a definitive result until sufficient evidence is available to reclassify it as either likely benign or likely pathogenic. Regular monitoring for updates on the variant's classification is crucial, as evidence from co-segregation studies in multi-generational families, reports of the same variant in well-characterized cases, or functional studies can aid in its reclassification. Clinicians can also collaborate with genetic experts through platforms like GeneMatcher to re-evaluate variant classifications over time as new evidence emerges.

Another complex scenario arises when genetic findings indicate susceptibility to disease rather than causation of a Mendelian disorder. As previously mentioned, *APOL1* risk variants, G1 and G2, are strongly associated with increased risk of FSGS and other kidney diseases, particularly in individuals of African ancestry [35]. However, the presence of these *APOL1* risk variants does not invariably lead to disease, as environmental and other genetic factors modulate risk and many healthy individuals are homozygous or compound heterozygous for *APOL1* risk variants. Being G1/G1, G2/G2 or G1/G2 carries implications for affected individuals and their family members, as these findings can inform personalized monitoring strategies but should not be equated with a definitive genetic diagnosis. It is crucial to clearly communicate the distinction between genetic susceptibility and Mendelian inheritance to patients and their families to prevent undue anxiety or unnecessary medical interventions.

A negative result does not exclude the possibility of a monogenic cause for glomerular disease. Technical limitations, such as difficulties in detecting structural variants or variants in non-coding regions, may contribute to negative results. In cases with a strong clinical suspicion of a genetic cause, further testing methods, such as whole-genome sequencing or long-read sequencing, should be considered. Additionally, periodic reanalysis of genomic data is beneficial as advancements in variant-calling algorithms or new gene discoveries may later identify previously undetectable variants. Other inheritance models, such as mitochondrial or polygenic inheritance, or even non-genetic causes, should also be explored to fully understand the patient's condition.

### Benefits of genetic testing

Accurate diagnosis is critical for all conditions, but it holds particular importance in glomerular diseases. In the absence of a genetic or precise histological diagnosis, patients with proteinuria are likely to receive corticosteroids and immunosuppressive drugs, which carry significant side effects. A positive genetic test can prevent unnecessary treatment, allowing management with antiproteinuric agents alone.

Genetic testing is increasingly recognized as a critical tool in kidney transplantation, with growing implications for the evaluation and management of both transplant recipients and living donors. It offers several benefits as it identifies patients needing heightened surveillance or targeted interventions for extrarenal manifestations and reassures when recurrence risk is low. For living donors, it is pivotal in evaluating related donors, informing eligibility decisions, and safeguarding their long-term health by detecting predisposition to kidney dysfunction [26]. For most genetic glomerular diseases, disease recurrence is uncommon; however, rare immune responses may occur. For instance, anti-nephrin antibodies have been reported in Finnish-type nephrotic syndrome [43, 44], and anti-GBM antibodies have been observed in rare cases of Alport syndrome [45]. These responses represent immune reactions to unfamiliar proteins rather than true disease relapses.

And, of course, the benefit of a precise diagnosis includes those that are applicable to any genetic condition, as shown in Table 3.

**Table 3:** Benefits of a positive genetic test in glomerular diseases.

• Avoid/stop immunosuppressive therapy.
• Offer prognostic information.
• Examine extrarenal features.
• Provide genetic counseling.
• Allow participation in new trials for genetic glomerulopathies.
• Obviate the need for kidney biopsy (exception: *COL4A3/4* heterozygous variant).
• Diagnosis of relatives.
• Allow the use of gene-specific therapies that are available

## CONCLUSION

In summary, integrating genetic testing into the diagnostic process for glomerular diseases enhances diagnostic accuracy, especially in cases with inconclusive kidney biopsy results. While kidney biopsy remains essential, genetic testing can clarify the etiology of conditions like FSGS, helping tailor treatment by identifying when immunosuppressive therapies may be unnecessary. Genetic results require careful interpretation, as positive findings may pinpoint a monogenic cause, while variants of uncertain significance need ongoing evaluation. Negative results do not rule out genetic causes, suggesting further testing may be necessary. Overall, a clinicopathological approach, enriched by genetic testing, offers a precise framework for diagnosis and management in glomerular diseases. Future directions for research and clinical practice include potential advancements in genetic testing and personalized medicine, which could further improve diagnostic precision and individualized treatment strategies.

## Data Availability

No new data were generated or analyzed in support of this research.
